# Requirement for Ergosterol in V-ATPase Function Underlies Antifungal Activity of Azole Drugs

**DOI:** 10.1371/journal.ppat.1000939

**Published:** 2010-06-03

**Authors:** Yong-Qiang Zhang, Soledad Gamarra, Guillermo Garcia-Effron, Steven Park, David S. Perlin, Rajini Rao

**Affiliations:** 1 The Johns Hopkins University School of Medicine of Baltimore, Maryland, United States of America; 2 Public Health Research Institute, New Jersey Medical School-UMDNJ, Newark, New Jersey, United States of America; University of Toronto, Canada

## Abstract

Ergosterol is an important constituent of fungal membranes. Azoles inhibit ergosterol biosynthesis, although the cellular basis for their antifungal activity is not understood. We used multiple approaches to demonstrate a critical requirement for ergosterol in vacuolar H^+^-ATPase function, which is known to be essential for fungal virulence. Ergosterol biosynthesis mutants of *S. cerevisiae* failed to acidify the vacuole and exhibited multiple *vma* ^−^ phenotypes. Extraction of ergosterol from vacuolar membranes also inactivated V-ATPase without disrupting membrane association of its subdomains. In both *S. cerevisiae* and the fungal pathogen *C. albicans*, fluconazole impaired vacuolar acidification, whereas concomitant ergosterol feeding restored V-ATPase function and cell growth. Furthermore, fluconazole exacerbated cytosolic Ca^2+^ and H^+^ surges triggered by the antimicrobial agent amiodarone, and impaired Ca^2+^ sequestration in purified vacuolar vesicles. These findings provide a mechanistic basis for the synergy between azoles and amiodarone observed *in vitro*. Moreover, we show the clinical potential of this synergy in treatment of systemic fungal infections using a murine model of Candidiasis. In summary, we demonstrate a new regulatory component in fungal V-ATPase function, a novel role for ergosterol in vacuolar ion homeostasis, a plausible cellular mechanism for azole toxicity in fungi, and preliminary *in vivo* evidence for synergism between two antifungal agents. New insights into the cellular basis of azole toxicity in fungi may broaden therapeutic regimens for patient populations afflicted with systemic fungal infections.

## Author Summary

Systemic fungal infections impose a significant threat to public health and therapeutic options to treat these diseases remain limited. Azoles represent the largest category of anti-fungal drugs and repress fungal growth by inhibiting biosynthesis of ergosterol, an important constituent of fungal membranes. Despite the wide use of azoles in the clinic for decades, the cellular basis for their antifungal mechanism remains elusive. In this study, we use a range of genetic, cellular and biochemical approaches to reveal a requirement for ergosterol in vacuolar H^+^-ATPase function. V-ATPase plays essential roles in diverse cellular processes, and is required for fungal virulence. Concomitant ergosterol feeding restores vacuolar acidification and growth in cells treated with fluconazole. These results suggest that the critical requirement for ergosterol in V-ATPase function may underlie the antifungal activity of azoles. Moreover, we show in a mouse Candidiasis model that combining an ion homeostasis-disruptive drug with azole is an effective approach to treat fungal infections.

## Introduction

Pathogenic fungal species, including *Aspergillus*, *Candida*, *Histoplasma* and *Cryptococcus* among others, cause infections ranging from mucocutaneous disorders to life-threatening invasive diseases that can involve any organ. In the past two decades, expanding populations of immunocompromised patients and increased use of invasive devices and implants have led to an increase in the incidence of fungal infections [1], [2]. Currently, four major categories of antifungal therapeutics are available to treat invasive fungal infections: polyenes, azoles, echinocandins and flucytosine [3]. Azole drugs are the most widely deployed in clinics, and inhibit the biosynthesis of ergosterol, the fungal-specific sterol. The primary molecular target of azole drugs is Erg11p (Entrez GeneID: 856398), a P450 cytochrome that catalyzes 14α-demethylation of lanosterol in the ergosterol biosynthesis pathway [4]. Besides azoles, a number of other drugs such as allylamines and morpholines used in medicine and agriculture also inhibit ergosterol biosynthesis [5], [6].

Ergosterol is an important constituent of membrane lipids, similar to vertebrate cholesterol, and modulates the fluidity, permeability and thickness of the membrane. These sterols preferentially associate with sphingolipids in microdomains that have been postulated to have important roles in membrane organization and function [7], [8]. Ergosterol is most abundant in the plasma membrane and has been implicated in several cellular processes including sporulation, pheromone signaling and plasma membrane fusion during mating and endocytosis [9], [10]. Discernable amounts of ergosterol have also been found in membranes of intracellular organelles including peroxisomes, mitochondria, vacuoles and ER [11]. Some studies have ascribed a regulatory role at these intracellular compartments, including homotypic vacuole fusion [12], mitochondrial biogenesis and inheritance, and protein sorting along exocytosis and endocytosis pathways [13], [14]. The absence of ergosterol in mammals and suppression of fungal proliferation by a battery of ergosterol biosynthesis inhibitors emphasize the importance and utility of ergosterol as an effective target in antifungal chemotherapy. Yet, despite nearly two decades of use and the general recognition of the importance of ergosterol to fungal cells our understanding of the specific cellular processes disrupted by ergosterol deprivation following azole therapy remains minimal.

The limited categories of antifungal agents and emergence of resistance to existing antimycotics have prompted a search for compounds with alternative modes of action. The anti-arrhythmia drug, amiodarone, was recently documented to exhibit fungicidal activity [15], [16]. This cationic amphipathic compound inserts into the lipid bilayer where it elicits membrane hyperpolarization, and influx of H^+^ and Ca^2+^ into the cytoplasm [15], [17]. Within minutes, amiodarone also elicits a transcriptional response to starvation and blocks cell cycle progression [18]. A screen of the yeast haploid deletion library for amiodarone hypersensitivity revealed multiple *vma* genes encoding subunits of the vacuolar membrane H^+^-ATPase [15]. The V-ATPase is critical for generation of a pH gradient that drives secondary transporters to maintain cellular ion homeostasis. Since the fungicidal activity of amiodarone appears to be tightly coupled to ion stress [19], hypersensitivity of *vma* mutants was ascribed to defects in ion homeostasis. Notably, deletion mutants of several *erg* genes in the ergosterol biosynthesis pathway were also identified in the screen [15], although the underlying mechanism for their amiodarone hypersensitive phenotype was unclear. Meanwhile, a screen of the yeast haploid deletion mutant library for strains with alterations in vacuolar pH revealed that *erg* mutants, like *vma* mutants, had severely alkaline vacuoles (Brett, C.L., Rao. R. *et al.*, unpublished data). A separate study showed that sphingolipid, the other major membrane lipid component found associated with ergosterol in detergent-resistant microdomains, was required for the structural integrity of V-ATPase domains [20]. In light of these observations, we investigated a potential link between ergosterol and V-ATPase function, which led to a mechanistic basis for the antifungal activity of azole drugs. As a first step in exploiting our observations for improved management of invasive fungal diseases, we assessed the efficacy of combining fluconazole with ion homeostasis-disruptive agent amiodarone in a murine Candidiasis model.

## Results

### Mutants defective in ergosterol biogenesis exhibit multiple *vma^−^* phenotypes

**Figure 1:**
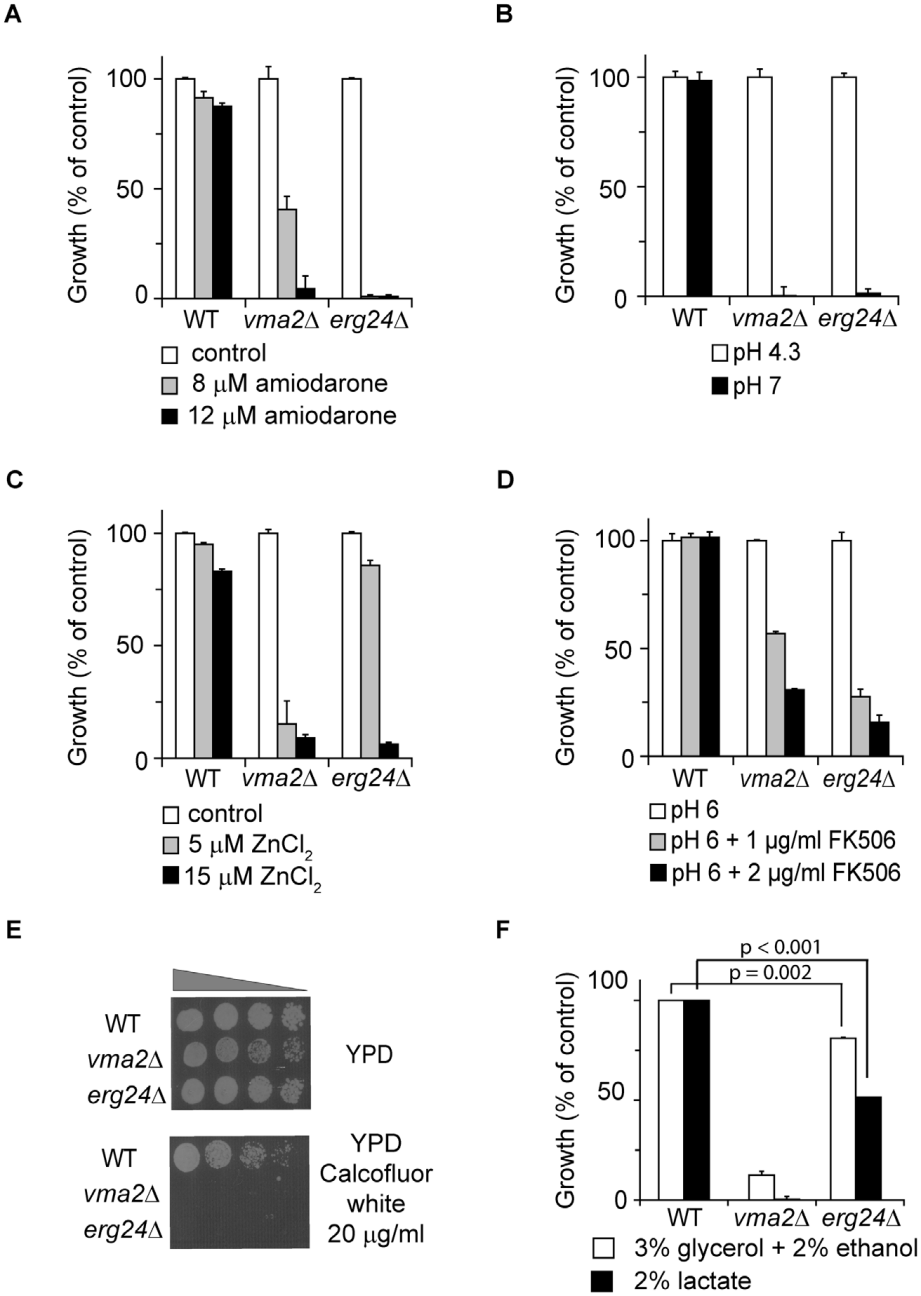
*erg24Δ* exhibits multiple *vma^−^* phenotypes. Growth of WT, *vma2Δ*, *erg24Δ* strains under different conditions. Values were normalized to growth of each strain under control condition (A and C), to growth of each strain at pH 4.3 (B), to growth of each strain at pH 6 without FK506 (D), or to growth of WT under the two conditions with non-fermentable carbon source (F). (E) Growth of the strains in YPD or YPD supplemented with Calcofluor white. All measurements were in triplicate, and means and standard deviations are plotted. *p* values of two-tailed t-test are shown.

A genome-wide screen of the *S. cerevisiae* haploid deletion collection for hypersensitivity to the antifungal agent amiodarone revealed multiple *vma* and *erg* mutants [15]. Given our previous observation that amiodarone triggered Ca^2+^ and H^+^ influx leading to fungal death from ion stress [15], [19] and the importance of V-ATPase in ion homeostasis, we considered the possibility that ergosterol may be important for V-ATPase function. A systematic examination of viable *erg* null mutants (*erg2Δ* [Entrez GeneID: 855242], *erg3Δ* [Entrez GeneID: 850745], *erg6Δ* [Entrez GeneID: 855003] and *erg24Δ* [Entrez GeneID: 855441]) revealed multiple *vma^−^* phenotypes, with *erg24Δ* displaying the most severe defects. In addition to hypersensitivity to amiodarone (Fig. 1A), *erg24Δ* was unable to grow at alkaline pH (Fig. 1B), a defining phenotype of *vma* mutants indicative of the inability to acidify vacuoles. Furthermore, *erg24Δ* exhibited hypersensitivity to Zn^2+^ toxicity and to the calcineurin inhibitor FK506, consistent with broad ion homeostasis defects characteristic of *vma* mutants (Fig. 1C–D). Yeast strains defective in trafficking of chitin synthase, including *vma* mutants, are more sensitive to toxicity from calcofluor white, an antimicrobial agent that binds to cell wall chitin. We showed that *erg24Δ* shared calcofluor white hypersensitivity with *vma2Δ* (Fig. 1E). Poor growth of *vma* mutants on high concentrations of non-fermentable carbon sources has been ascribed to oxidative stress from respiration. Although *erg24Δ* was able to grow on non-fermentable carbon sources, growth was significantly impaired (Fig. 1F). Overall, the novel observation that ergosterol biogenesis mutants largely phenocopy *vma* mutants suggests that cellular ergosterol content may be important for the function of V-ATPase.

### Ergosterol is critical for V-ATPase function

**Figure 2:**
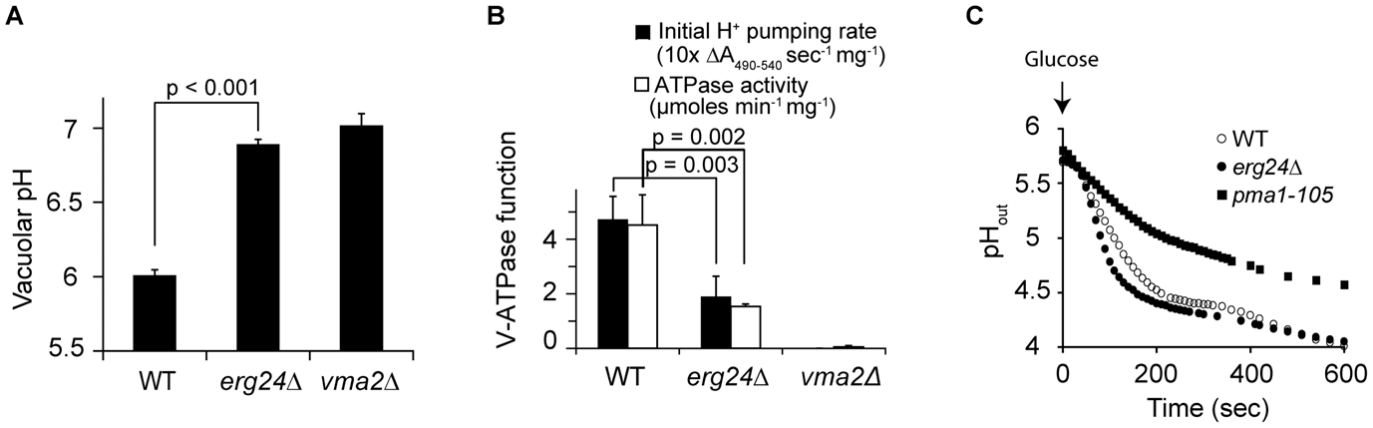
ERG24 deletion impairs the function of V-ATPase but not Pma1p. (A) Vacuolar pH of WT, *erg24Δ* and *vma2Δ* strains measured with pH sensitive fluorophore BCECF-AM, which accumulates in the yeast vacuole. (B) Initial H^+^ pumping rate was calculated from ΔA_490–540_ during the first 60 s after initiating the reaction. ATPase activity was calculated from drop of A_340_ between 3 and 6 minutes after initiating the reaction. Means and standard deviations are plotted from data for at least three independent vacuolar vesicle preparations. p values of two-tailed t-test are shown. (C) Medium acidification by Pma1 upon glucose activation was measured as described in Methods. Extracellular pH (pH_out_) was recorded after glucose was added to 2% at time 0.

V-ATPase hydrolyzes ATP and acidifies vacuolar compartments. To assess a possible requirement for ergosterol in V-ATPase function, we first measured the vacuolar pH in *erg* mutants using the pH-sensitive fluorescent dye BCECF. The acetoxy methyl ester of BCECF is taken up by cells and de-esterified in the vacuole where it accumulates [21]. While the vacuolar pH of wild-type cells was 6.0, vacuoles of *vma2Δ* (Entrez GeneID: 852424) cells were significantly more alkaline, around pH 7, as would be expected for loss of proton pump capacity (Fig. 2A). Vacuolar pH of all viable *erg* mutants closely resembled that of the *vma* mutant, as shown for *erg24*Δ (Fig. 2A). Next, we purified intact vacuolar vesicles from wild type, *erg24Δ* and *vma2Δ* strains, and compared V-ATPase function, including rates of proton pumping and ATP hydrolysis. There was no V-ATPase activity detectable in *vma2*Δ vacuoles as expected, whereas *in vitro* ATPase and H^+^ pumping activity were both diminished to about 40% of wild type levels in *erg24*Δ (Fig. 2B). Examination of sterol profiles in purified vacuoles confirmed the presence of ergosterol in wild type vacuoles and its absence in *erg24*Δ (not shown). Taken together, these results provide evidence for a role for ergosterol in V-ATPase activity.

The P-type H^+^-ATPase Pma1 (Entrez GeneID: 852876) has been documented to associate with ergosterol enriched domains [22], [23]. Upon glucose activation, it pumps protons out of cells to acidify the extracellular medium. To assess the effect of ergosterol depletion on Pma1 function, we examined extracellular acidification upon glucose activation in *erg24Δ* cells. As shown in Fig. 2C, the kinetics of medium acidification was substantially similar between the wild type and *erg24Δ*, while a previously characterized PMA1 mutant, *pma1-105*, reported to have a 65% reduction in activity, exhibited slower acidification rate as expected [24]. These data suggest that ergosterol is not required for Pma1 function and support the specificity of the ergosterol effect on V-ATPase.

**Figure 3:**
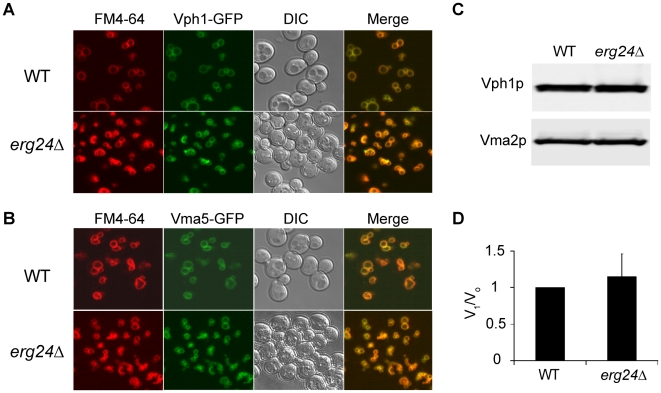
Expression and localization of V-ATPase in *erg24Δ*. Vph1p (A) and Vma5p (B) were tagged with C-terminal GFP at their chromosomal loci in WT and *erg24Δ* strains. Vacuolar membranes were stained with FM4-64 for 30 min in YPD and chased for another 30 min with fresh YPD. (C) Immunoblotting of Vph1p and Vma2p in vacuolar vesicles isolated from WT and *erg24Δ*. (D) Vma2p to Vph1p ratio in WT was designated as V_1_/V_o_ ratio of 1. Calculation was based on data from three independent vacuolar vesicle preparations for each strain. Means of the ratios and standard deviation are plotted.

To investigate the mechanism underlying the requirement of ergosterol for optimal V-ATPase function, we first asked if V-ATPase localization was altered in the *erg24*Δ mutant. The V-ATPase is made up of 14 subunits organized into the V_o_ sector, integral to the membrane, and the cytoplasmic V_1_ sector that reversibly dissociates from the membrane [25]. Figure 3A & 3B show that representative subunits from the V_o_ sector (Vph1-GFP) and the V_1_ sector (Vma5-GFP) colocalized with the vacuolar membrane stain (FM4-64) in *erg24Δ* cells, similar to the isogenic wild type. It was possible that the ergosterol biogenesis defect significantly decreased V-ATPase expression or caused the dissociation of V_1_ from V_o_ domain. However, analyses of representative V-ATPase subunits in vacuolar vesicles purified from the wild type and *erg24Δ* showed similar expression levels and V_1_/V_o_ ratios that were identical between the two strains (Fig. 3C & 3D).

**Figure 4:**
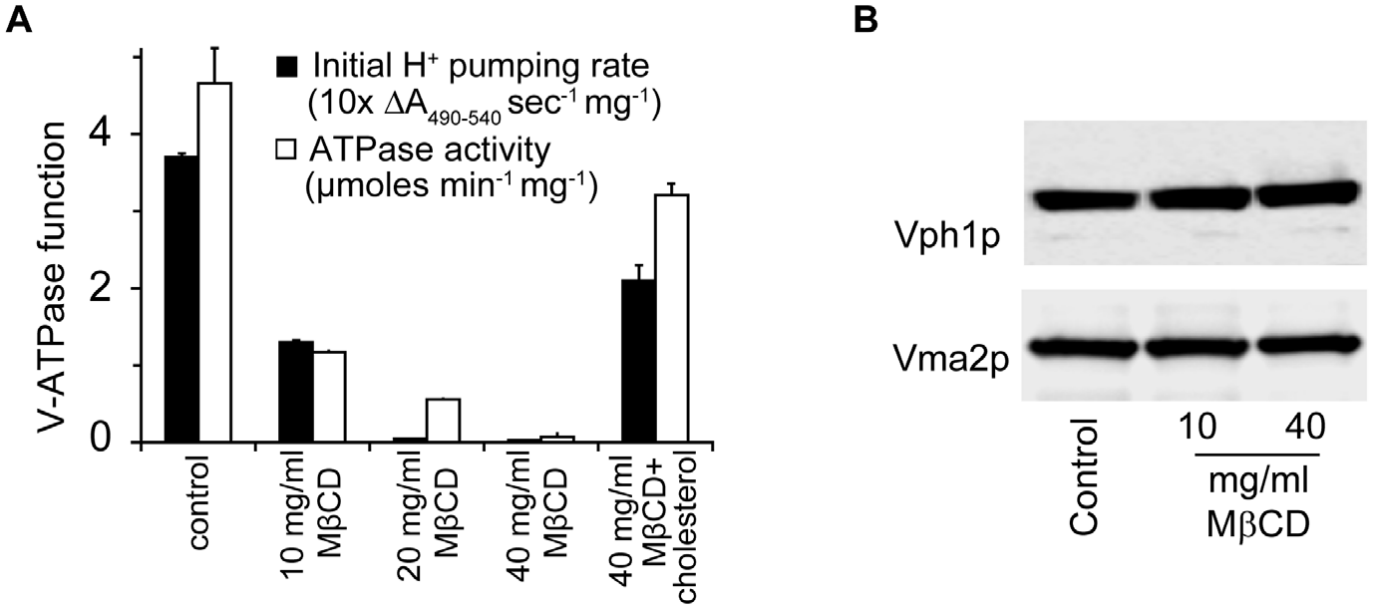
Ergosterol is critical for V-ATPase function. (A) WT vacuolar vesicles were incubated with MβCD or MβCD preloaded with cholesterol (cholesterol to MβCD ratio 1∶20 by weight) at 4°C for 30 min. Vacuolar vesicles were spun down and analyzed for ATPase function. (B) WT vacuolar vesicles treated with MβCD for 30 min at 4°C were spun down and used for immunoblotting to assess the abundance of Vph1p and Vma2p.

The oligosaccharide Methyl-β-Cyclodextrin (MβCD) extracts sterols from cellular membranes. We observed a dose dependent loss of V-ATPase activity following treatment of purified vacuolar vesicles with MβCD, which could be blocked by preloading cholesterol into MβCD (Fig. 4A). Both ATP hydrolysis rates and H^+^ pumping declined at similar rates, suggesting an inhibition of the intact V_1_V_o_ complex. This was verified by immunoblot analysis of vacuolar membranes collected by centrifugation after MβCD treatment (Fig. 4B): we did not observe a loss of either V_1_ (Vma2p) or V_o_ (Vph1p) subunits, suggesting that the two sectors remain associated after ergosterol extraction. In contrast, a previous study pointed to a role for sphingolipids in maintaining structural integrity of the V-ATPase enzyme complex [20]. We conclude that ergosterol constitutes a critical component in the lipid membrane environment for V-ATPase function.

### The antifungal drug fluconazole disrupts V-ATPase function

Azole drugs exert their fungistatic effect by inhibiting ergosterol biosynthesis, specifically targeting lanosterol demethylase (Erg11p), which is the enzymatic step immediately upstream from Erg24p. Despite the wide spread use of azole antifungals, our understanding of the specific cellular pathways disrupted by azoles is limited. In addition to ergosterol depletion, fluconazole treatment results in accumulation of lanosterol (substrate of Erg11p) and its derivative 14-methyl-3,6 diol [26]. Based on analysis of sterol profiles in fluconazole susceptible and resistant strains, it was concluded that 14-methyl-3,6 diol toxicity [26], [27] was responsible for azole-mediated growth arrest. To specifically assess the functional effect of ergosterol depletion following azole treatment, we used the *S. cerevisiae* strain WPY361 with a gain-of-function mutation in *UPC2* (Entrez GeneID: 851799), *upc2-1*, that allows overexpression of ATP-binding cassette transporters required for uptake of exogenously added sterol under aerobic condition [28], [29]. Table 1 shows that growth inhibition caused by fluconazole treatment in WPY361 can be reversed by exogenous supply of ergosterol. To examine whether ergosterol feeding represses endogenous sterol metabolism and reduces accumulation of intermediates and derivatives, we analyzed sterol profiles in *upc2-1* cells after six hours of exposure to fluconazole and exogenous ergosterol. As expected, fluconazole alone caused reduction of ergosterol content (nine-fold) and accumulation of lanosterol and a major derivative, likely to be 14-methyl 3,6-diol (Fig. S1). Compared with cells treated with fluconazole alone, cells treated with fluconazole and ergosterol had three-fold higher ergosterol content, yet the level of lanosterol and its derivatives remained the same (Fig. S1). These data indicate that ergosterol feeding did not reduce accumulation of intermediates and derivatives. Thus, depletion of ergosterol, rather than the toxicity of intermediates and derivatives, is a plausible mechanism for the antifungal activity of fluconazole.

**Table 1:** Ergosterol feeding relieves growth inhibition by fluconazole.

Treatment	Ab600
Control	1.04±0.01
Control + ergosterol	0.96±0.06
5 µg/ml FLC	1.00±0.02
5 µg/ml FLC + ergosterol	0.94±0.05
25 µg/ml FLC	0.05±0.01
25 µg/ml FLC + ergosterol	0.96±0.01
100 µg/ml FLC	0.04±0.02
100 µg/ml FLC + ergosterol	0.88±0.03

WPY361 (*upc2-1* mutant) was grown in YPD containing fluconazole (FLC), ergosterol, or a combination for 30 hours in a 96-well plate at 30°C in quadruplicate. Average of Absorbance values at 600 nm (Ab600) and standard deviations are shown. Ergosterol stock (5 mM in Tween∶ethanol [1∶1]) was added for a final concentration of 50 µM. Equal volume of Tween∶ethanol was added to samples without ergosterol as control.

**Figure 5:**
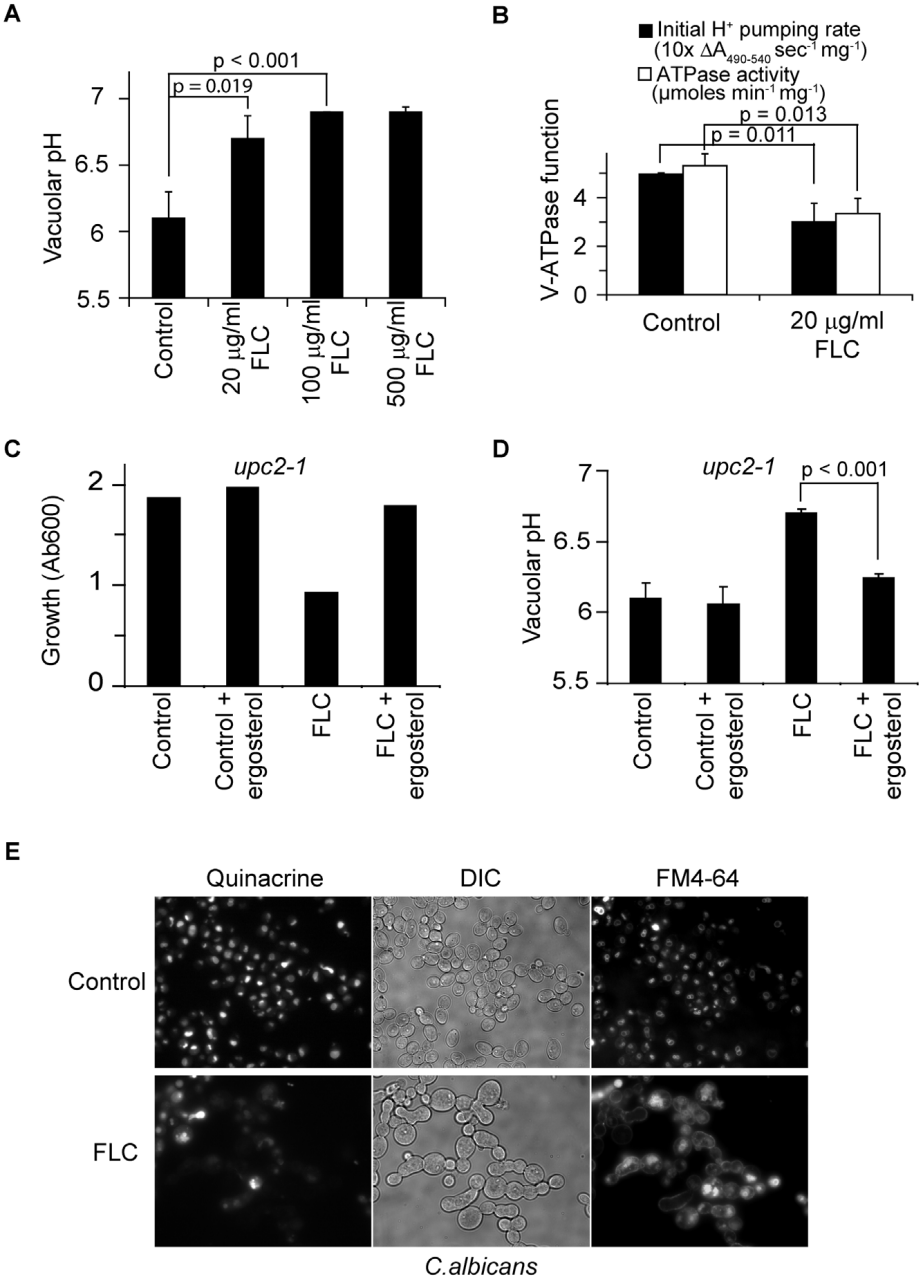
Fluconazole treatment disrupts V-ATPase function. (A) Vacuolar pH of WT (BY4742) cultures treated with fluconazole at specified concentrations for 6 hours in YPD. (B) Proton pumping rate and ATPase activity of WT (BY4742) cultures treated with or without fluconazole for 6 hours in YPD. Three independent batches of vacuolar vesicles were isolated and used to assay V-ATPase function. Cultures of *upc2-1* mutant, WYP361, were treated with fluconazole (100 µg/ml), ergosterol (50 µM) or their combination at OD 0.1. Growth (C, representative of three independent experiments) was assessed at 8 hour post-treatment, and vacuolar pH (D) was assessed at 6 hour post-treatment. (E) WT *C. albicans* cells (SC5314) were grown in YPD with or without fluconazole for 5 hours. FM4-64 was added to the cultures to stain vacuoles for 30 min. Cells were chased with fresh YPD with or without fluconazole for 20 min followed by quinacrine addition for another 5 min. Fluorescence microscopic images of the cells were taken immediately after washing. Means and standard deviations are plotted. p values of two-tailed t-test are shown.

Based on our evidence that ergosterol was required for optimal V-ATPase function, we predicted that azole treatment would similarly impair activity of the V-ATPase. Indeed, we show that fluconazole treatment resulted in a dose-dependent alkalinization of vacuolar pH, consistent with depletion of ergosterol from the vacuolar membrane (Fig. 5A). Furthermore, vacuolar membrane vesicles purified from fluconazole treated cells showed significant reductions in both H^+^ pumping rates and ATPase activity (Fig. 5B). Next, we evaluated the effect of ergosterol feeding on vacuolar pH in the *upc2-1* mutant. Not only did exogenous ergosterol restore growth in fluconazole treated cells, vacuolar acidification closely resembled that of untreated cells (Fig. 5C & 5D). This correlation strengthens the hypothesis that V-ATPase inhibition contributes to the cellular mechanism of azole activity.

To extend these observations in the human pathogen *Candida albicans*, we monitored vacuolar uptake of the fluorescent weak base quinacrine. Previous studies have demonstrated pH-dependent vacuolar accumulation of quinacrine, which was abolished in the homozygous *vma7^−/−^* mutant [30]. We observed robust quinacrine fluorescence in *C. albicans* vacuoles, colocalizing with FM4-64 staining of vacuolar membranes. Fluconazole treatment drastically reduced vacuolar accumulation of quinacrine in most cells, indicative of impaired vacuolar acidification (Fig. 5E). Additionally, trafficking of FM4-64 to the vacuolar membrane was impaired, consistent with endocytosis defects seen in *vma* mutants [30]. We note that following 6 h of fluconazole treatment, cells failed to divide but continue to increase in size, as previously reported [9]. These data suggest that the requirement of ergosterol for V-ATPase function is conserved in fungi. Given the importance of V-ATPase function and vacuolar acidification in diverse cellular processes, we conclude that disruption of V-ATPase function plays a critical role in antifungal activity of azole drugs. Consistent with this conclusion, both *vma7^−/−^* and *erg24^−/−^*mutants of *C. albicans* exhibit defective virulence in murine models of Candidiasis [30], [31].

### Impairment of V-ATPase function by azoles underlies synergism with amiodarone

We showed previously that amiodarone triggered a cytosolic H^+^ and Ca^2+^ surge in the baker's yeast and that mutants defective in ion homeostasis were hypersensitive to amiodarone toxicity [15]. Given the pivotal role of the V-ATPase in maintaining intracellular cation homeostasis, we predicted that ergosterol depletion by azole treatment would impair V-ATPase function and exacerbate disruption of cation homeostasis by amiodarone.

**Figure 6:**
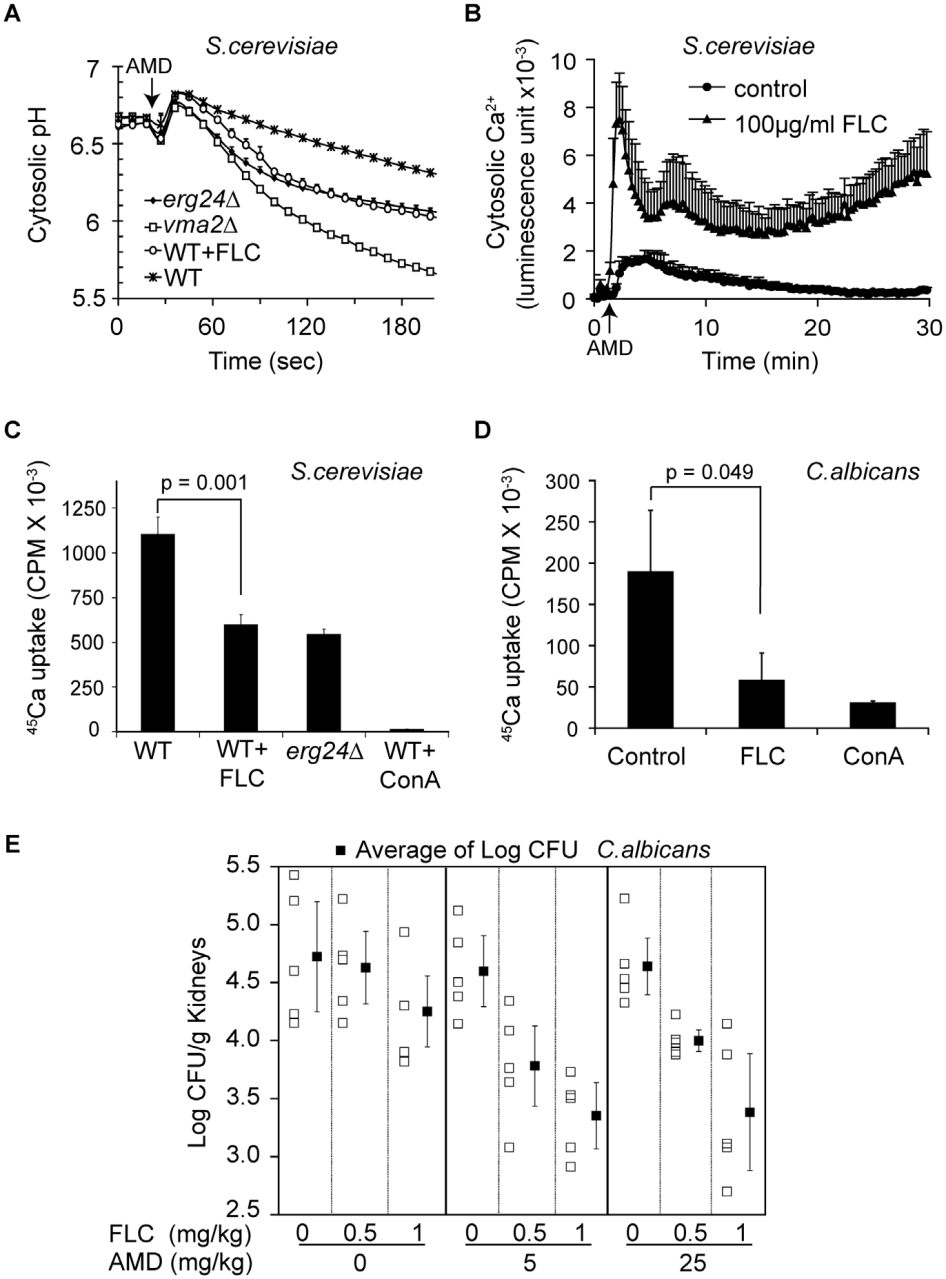
Synergy between fluconazole and amiodarone. (A) Early log phase cells expressing pHluorin were grown for 6 hours to mid log phase (OD ∼1) in SC minus leucine medium with or without fluconazole. Amiodarone (10 µM) was injected at the *arrow*. Measurement was done in triplicate, and the means and standard deviations are plotted [32]. (B) Early log phase WT cells expressing aequorin were grown with or without fluconazole for 4 hours. Amiodarone (10 µM) was injected at the *arrow*. Ca^2+^-dependent Aequorin luminescence was measured in triplicate as previously described [15]. For ^45^Ca uptake, early log phase WT cells of *S. cerevisiae* (C) and *C. albicans* (D) were grown with or without fluconazole (20 µg/ml for *S. cerevisiae*, 1 µg/ml for *C. albicans*) for 6 hours. Vacuolar vesicles isolated from these cultures were assayed for MgATP-dependent ^45^CaCl_2_ uptake as described in Materials and Methods [33]. Concanamycin A was added to assess V-ATPase dependence of CaCl_2_ uptake. p values of two-tailed t-test were shown. (E) Mice infected with *C. albicans* (ATCC 36082) were treated with vehicle, fluconazole, amiodarone, and their combination intraperitoneally. On day 4 post-infection, mouse kidneys (5 per group) were removed, weighed, homogenized, serially diluted and then plated onto YPD agar plates containing chloramphenicol and ampicillin. CFU were counted after 48 hours. Means of CFU per gram of kidney under each treatment and standard errors are plotted.

We first tested this hypothesis in the *S. cerevisiae* model. We used pH sensitive GFP (pHluorin) to monitor changes in cytosolic pH in wild type, *vma2Δ* and *erg24Δ* strains upon exposure to amiodarone [32]. Cytosolic acidification was most pronounced in the *vma2Δ* mutant consistent with a loss in the ability to transport H^+^ from the cytosol to the vacuole. Depletion of ergosterol in *erg24Δ* mutant or by fluconazole treatment also exacerbated cytosolic acidification, relative to wild type, upon amiodarone addition (Fig. 6A). We have previously demonstrated defective clearance of cytosolic Ca^2+^ in *vma* mutants following exposure to amiodarone [15]. We now show that pretreatment with fluconazole exacerbates the cytosolic Ca^2+^ surge elicited by amiodarone, consistent with defective V-ATPase function (Fig. 6B).

The proton gradient established by the V-ATPase drives vacuolar sequestration of excessive cytosolic Ca^2+^ by H^+^/Ca^2+^ exchange mechanisms. The importance of the V-ATPase is demonstrated by the inability of purified vacuolar vesicles to sequester ^45^Ca^2+^ following treatment with the V-ATPase inhibitor concanamycin A (Figure 6C). As predicted by our hypothesis, vacuolar vesicles purified from *erg24Δ* or fluconazole-treated wild-type *S. cerevisiae* both showed similar impairment in their ability to sequester Ca^2+^ (Fig. 6C). Likewise, vacuolar vesicles purified from *C. albicans* cells treated with fluconazole were also impaired in sequestering Ca^2+^ (Fig. 6D). Thus, our observations provide a mechanistic basis for previous reports of synergism between azoles and amiodarone against pathogenic fungi *in vitro* [15].

To investigate the potential clinical application of this synergism, we studied the effect of combining fluconazole and amiodarone in a murine candidiasis model. The microbial burden of *Candida albicans* in kidneys was assessed 3 days following intravenous infection of Balb/C mice. AMD treatment doses were presented at 5.0 and 25 mg/kg, while FLC doses were 0.5 and 1 mg/kg with treatments given once daily for three days after the first dose. In the absence of amiodarone, there was a significant dose-dependent effect of FLC on *C. albicans* (ratio of geometric means of the cfu count per 1 mg/kg of FLC was 0.5914, p = 0.01). In the absence of FLC, amiodarone did not confer a significant antifungal activity at the concentrations tested (ratio of geometric means of the cfu count per 1 mg/kg of amiodarone was 0.9996, p = 0.95). Yet in combination with fluconazole, the two doses of amiodarone significantly reduced *C. albicans infection* above and beyond the dose-dependent effect of fluconazole (ratio of geometric means of CFU with amiodarone versus without, for the same dose FLC = 0.4969, p<0.001) (Fig. 6E). These data provide proof of principle that combining azole drugs with other antifungal compounds that disrupt intracellular cation homeostasis could be a promising therapeutic strategy to treat systemic fungal infections.

## Discussion

In fungal cells, V-ATPase acidifies intracellular compartments including the vacuole, endosomes, and late-Golgi. Mutants lacking V-ATPase exhibit characteristic phenotypes of growth sensitivity to alkaline pH, calcofluor white, Ca^2+^ and metal ion stress, and are unable to grow on high concentrations of non-fermentable carbon sources [25]. We show that mutants defective in ergosterol biosynthesis exhibit most of these characteristic *vma^−^* phenotypes. Furthermore, we showed a reduction of vacuolar acidification by ergosterol depletion, restoration of vacuolar acidity by ergosterol feeding, and used biochemical assays of H^+^ pumping with purified vacuolar vesicles to collectively demonstrate the requirement of ergosterol for optimal V-ATPase function. This functional link explains simultaneous identification of multiple ergosterol biosynthesis genes (*erg*) and V-ATPase subunit genes (*vma*) in a number of genome-wide screens, including sensitivity to low Ca^2+^, alkaline stress, and acid stress [33]–[35]. V-ATPase mutants and ergosterol biosynthesis mutants also share defects in endocytosis [9], [36]. Additionally, in pathogenic fungal species, both *vma* and *erg* mutants are avirulent [30], [31], [37]. Disruption of V-ATPase function by ergosterol deprivation provides a mechanistic basis for these similarities.

To investigate the underlying basis for the requirement of ergosterol in V-ATPase function, we first checked the localization and abundance of V-ATPase in *erg24Δ*. Fluorescent microscopy and immunoblot analysis ruled out possible mislocalization and reduced abundance of V-ATPase in *erg24Δ* vacuolar membrane. In yeast, the V-ATPase complex undergoes rapid reversible dissociation into non-functional V_1_ and V_o_ sectors in response to glucose withdrawal [38]. A study with Baby Hamster Kidney cells suggested that increasing ratio of Intact V_1_/V_o_ along the endocytic pathway effectively increased acidity along the compartments in the pathway [39]. Analyses of immunoblot results in this study show that V_1_ and V_o_ domains are still associated, and V_1_/V_o_ ratio remains unchanged upon ergosterol deprivation by treating cells with fluconazole and treating vacuolar vesicles with MβCD. These data rule out dissociation of V_1_ from V_o_ domain as the cause of reduced V-ATPase function, and indicate that ergosterol directly modulates the activity of V-ATPase. Sphingolipid, another major component of lipid raft, was thought to affect V-ATPase function by maintaining the structural integrity of V-ATPase because V_1_ subunits (Vma1p, Vma2p and Vma5p) dissociate from V_o_ domain during Ficoll gradient procedure in the sphingolipid mutant *sur4Δ*. In contrast, Vma2p remained associated with Vph1p after Ficoll gradient procedure in *erg24Δ* mutant and after ergosterol extraction by MβCD in the wild type. Thus, these two key membrane lipid components play distinct roles in maintaining V-ATPase function.

The precise molecular basis of V-ATPase regulation by ergosterol remains to be determined. In mammalian cells, V-ATPase has been shown to associate with cholesterol-rich microdomains, with loss of vesicular acidification reported upon treatment with β-methylcyclodextrin [40]. A number of studies showed that inhibitors could interact with lipid bilayer and affect V-ATPase function by restricting its structural flexibility [41], [42]. Mechanisms proposed to explain the regulation of Ca^2+^-ATPase and Na^+^,K^+^-ATPase by membrane lipids include lowering of free energy of activation and proper packing at protein-protein interfaces [43], [44]. Altered sterol compositions are known to affect membrane packing and rigidity: fluorescence anisotropy probes have revealed increased membrane fluidity and permeability upon fluconazole treatment [45], consistent with alterations in activity of membrane-localized pumps and transporters, and a critical role for ion homeostasis mechanisms in drug treated cells. It is also possible that ergosterol may affect V-ATPase function indirectly by modulating regulatory interactions with other proteins. The complex multi-subunit structure of V-ATPase and its intimate association with sterols and spingolipids indicate that sophisticated regulatory mechanisms must be in place to ensure proper assembly, configuration, and communication among these components. Ergosterol depletion may affect other membrane functions besides vacuolar acidification. The plasma membrane H^+^-ATPase, Pma1, is a major efflux mechanism for protons, and is also found associated with ergosterol-rich microdomains [22], [23]. However, we found that Pma1-mediated proton pumping function was not altered in *erg24Δ*, in contrast to the pronounced effect seen on the V-ATPase. This suggests that membrane proteins have specific lipid requirement for their functions.

While the molecular target of azole drugs, Erg11p, has been extensively characterized, not much is known about the cellular basis of fungal growth inhibition. Inhibition of Erg11p by fluconazole results in accumulation of lanosterol and its derivative 14-methyl-3,6 diol. In azole resistant *erg3* mutants, 14-methyl fecosterol accumulates upon treatment with fluconazole [26], [27]. This has led to the notion that toxicity of sterol derivatives such as 14-methyl-3,6 diol mediates the action of fluconazole [45], [46]. We exploited the ability of a recently described *upc2-1* mutation that allows uptake of ergosterol under aerobic conditions to distinguish between the effects of byproduct accumulation and ergosterol depletion on cell growth. The ability of exogenous ergosterol to reverse growth inhibition by fluconazole supports a plausible alternative hypothesis that antifungal activity of azoles is due to ergosterol depletion. Although we ruled out a corresponding decrease in lanosterol and other derivatives in the ergosterol fed cells, we cannot exclude the possibility that a specific ratio of ergosterol to other sterols may counter potential toxic effects. This possibility could be tested by varying ratios of sterols in *upc2-1* feeding experiments; however, 14-methyl-3,6 diol is not commercially available and its potential toxicity cannot be directly assessed at this time. Furthermore, our results warrant more careful examination of the role of 14-methyl fecosterol in the potential compensation of ergosterol function in membranes.

Some reports suggest a role for ROS in the toxicity of azoles to fungal cells [47]. It is worth noting that cellular ROS level in these studies was measured with the fluorescent dye DCFH-DA (2′,7′-dichlorofluorescin-diacetate) which has also been documented to respond to pH alterations [48]. Given the effect of azole drugs on pH homeostasis, the role of ROS in azole-induced growth inhibition may need to be re-evaluated.

The far-reaching effect of V-ATPase is illustrated by diverse phenotypes exhibited by V-ATPase mutants. By disrupting the function of V-ATPase through ergosterol deprivation, azole drugs can affect a wide range of cellular processes, including cation homeostasis, protein sorting, processing and degradation. Importantly, V-ATPase function and vacuolar processing of virulence factors are required for pathogenesis [30], [37]. Although we cannot preclude additional cellular targets of azole toxicity, disruption of V-ATPase function is sufficient to repress growth and attenuate fungal virulence and is likely to be a critical mechanism underlying antifungal activity of azole drugs.

Amiodarone exhibits antimicrobial activity against a wide range of fungi and protozoa through disruption of H^+^ and Ca^2+^ homeostasis [15], [16], [49]. Additionally, *in vitro* studies showed azoles were synergistic with amiodarone against fungal pathogens, *e.g. C. albicans* and *C. neoformans* [15]. Interestingly, amiodarone interacts with fluconazole synergistically against fluconazole-resistant clinical isolates of *A. fumigatus* and *C. albicans* [50], [51]. Moreover, the synergy of amiodarone and posaconazole has been shown on the protozoan *T. cruzi* both *in vitro* and *in vivo* [49]. Uncovering the mechanism underlying this synergism may provide insight guiding the design of more potent antifungal therapy.

Data in this study show that ergosterol is required for the optimal function of V-ATPase, a central player in maintaining H^+^ and Ca^2+^ homeostasis. Therefore, depletion of ergosterol would be expected to exacerbate the disruption of H^+^ and Ca^2+^ homeostasis upon amiodarone treatment. Indeed, upon ergosterol depletion either by *erg* mutations or by azole treatment, ^45^Ca uptake by purified vacuolar vesicles was reduced while cytosolic H^+^ and Ca^2+^ surges increased upon exposure to amiodarone. Thus, we conclude that disruption of V-ATPase function in maintaining cation homeostasis by azoles contributes to the synergy between azoles and amiodarone.

Recently, we demonstrated that a combination of amiodarone and fluconazole in *C. albicans* resulted in dampening of the transcriptional response to either drug alone [52]. This effect could potentially stunt cellular stress responses occurring downstream of drug toxicity and thereby contribute to synergistic effects of the drugs. These data reveal an additional mechanism contributing to the synergism that is distinct from the ion homeostasis defects presented here. We and others have argued that non-overlapping but complementary insights can be obtained from phenotype versus transcriptional profiling [18]. Thus, genes involved in membrane integrity and ion homeostasis such as *VMA* and *ERG*, determine phenotype of growth sensitivity to amiodarone. These genes tend to be constitutively expressed, have a non redundant function and represent pathways upstream of the transcriptional response. On the other hand, genes that are differentially regulated in response to drug appear to play a collective, rather than individual, response to adaptation to stress. Taken together, these mechanisms contribute to a more complete picture of the complex cellular response to drug toxicity. Finally, in this study, we evaluated the clinical potential of combining fluconazole and amiodarone in treating fungal infections in a murine Candidiasis model. The synergy demonstrated in this experiment is proof-of-principle that combining azoles with agents disruptive to cation homeostasis is a promising approach to better manage fungal infections.

## Materials and Methods

### Yeast strains, media, and reagents

*S. cerevisiae erg^−^* and *vma^−^* mutant strains are from MATα deletion library (Invitrogen, Carlsbad, CA). WPY361 (MATa upc2-1 ura3-1 his3-11,-15 leu2-3,-112 trp1-1) was kindly provided by Dr. Will Prinz (NIDDK). Yeast cells were grown in standard SC (synthetic complete) medium or YPD (yeast extract, peptone and dextrose) medium at 30°C with shaking at 250 rpm unless specified otherwise. Media with non-fermentable carbon source contains 1% Bacto-yeast extract, 2% Bacto-peptone, 3% glycerol (v/v) plus 2% ethanol (v/v), or 2% sodium lactate. Antibodies against Vph1p and Vma2p were purchased from Invitrogen (Carlsbad, CA) or provided by Dr. Patricia Kane (Upstate Medical University, New York).

### Plasmid construction and yeast genetic manipulation

Plasmid pZR4.1 with pHluorin gene under *TEF1* promoter was constructed to measure yeast cytosolic pH. Briefly, *TEF1* promoter sequence was amplified from BY4742 genomic DNA with primers *Xba*ITEF1 and *Bam*HITEF1, which incorporate restriction sites of *Xba*I and *Bam*HI. *CYC1* terminator sequence was amplified with primers *BamH*ICYC1 and *Eco*RICYC1, which incorporate restriction sites of *EcoR*I and *Bam*HI. pHluorin gene sequence was amplified from pCB190YpHc plasmid with primers *BamH*IPhluo and *Bam*HIPhluoR, which incorporate *Bam*HI restriction site. The amplicons were digested with corresponding restriction enzymes and ligated sequentially with the backbone of pYEplac181, resulting in the gene for pHluorin being flanked by *TEF1* promoter and *CYC1* terminator. pZR4.1 was transformed to yeast strains to monitor cytosolic pH change upon exposure to amiodarone.

Wild type (mating type a) Vph1-GFP and Vma5-GFP strains in which GFP was fused to the C-terminus of the two proteins were purchased from Invitrogen. The Vph1-GFP and Vma5-GFP fragments were amplified from these strains with primers Vph1L2 + Vph1R1, and Vma5L2 + vma5R1, respectively. The amplicons were transformed to *erg24*Δ to replace the endogenous *VPH1* and *VMA5* genes by homologous recombination. Primer sequences are available upon request.

### Ergosterol feeding and sterol analysis

Ergosterol (Sigma) was dissolved in Tween 80∶ethanol (1∶1) as 5 mM stock. Stocks of ergosterol, fluconazole or their combination were added at the same time to WPY361 cells in YPD. For growth assay, stationary cultures were grown in 96-well plates for 30 hours at 30°C. For vacuolar pH measurement and sterol analysis, log phase cells were treated with ergosterol and fluconazole for 6 hours. Total sterols were extracted from ∼5×10^7^ log-phase cells after washing twice with water and analyzed with an Agilent 6850 gas chromatograph with an HP-1 column and FID as described previously [53]. Retention times for cholesterol, ergosterol and lanosterol were determined using standards. Cholesterol was added to each sample to normalize extraction efficiency.

### Vacuolar vesicle purification, MβCD treatment and ^45^Ca uptake assay

Vacuolar vesicles were prepared as described previously except that 10% Ficoll, instead of 8% Ficoll, was used in the second ultracentrifugation step to facilitate purification of vesicles from *erg24Δ* and fluconazole treated cells [54]. For ^45^Ca uptake assay, vacuolar vesicles were incubated in reaction buffer containing 5 µM CaCl_2_ with tracer quantities of ^45^CaCl_2_. After 5 min of incubation, vacuolar vesicles were filtered onto nitrocellulose membranes. The filters were washed and processed for liquid scintillation counting. Concanamycin A was added to 0.5 µM to assess the dependence of ^45^Ca uptake by vacuolar vesicles. For MβCD treatment, vacuolar vesicles were incubated with MβCD or MβCD preloaded with cholesterol (cholesterol to MβCD ratio 1∶20 by weight, Sigma, C4951) for 30 min at 4°C with gentle shaking. Vesicles were spun down and suspended in buffer C with proteinase inhibitors (aprotinin 2 µg/ml, leupeptin 1 µg/ml, pepstatin 1 µg/ml, chymostatin 2 µg/ml) for V-ATPase function assays and SDS-PAGE [20], [54].

### V-ATPase function assays

ATP-dependent proton pumping was assayed by monitoring change of ΔA_490–540_ [36]. The initial H^+^ pumping rates were calculated from the absorbance change in the first 60 seconds. ATPase activity was assessed by an enzyme-coupled assay monitoring depletion of NADH through oxidation at 340 nm. ATPase activity was calculated based on the absorbance decrease between three and six minutes after initiating the reaction. Concanamycin A was added at a final concentration of 0.1 µM to assess the specificity of V-ATPase.

### Intracellular pH measurement

Cytosolic pH was measured using pHluorin, a pH-sensitive GFP [32], under *TEF1* promoter in plasmid pZR4.1. Early log phase cells harboring plasmid pZR4.1 were grown for 6 hours to mid log phase (OD ∼1) in SC minus leucine medium with or without fluconazole. Cells were collected by centrifugation and suspended in SC to OD 3 in a 96-well plate, with 200 µl culture in each well. Amiodarone (stock 300 µM in H_2_O) was injected to give the specified final concentration. Fluorescence emission at 520 nm was measured in triplicate with excitation at 485 nm and 410 nm in a Fluostar Optima plate reader. Cytosolic pH was calculated based on the ratio of emission at 520 nm excited at 485 nm and 410 nm against a calibration curve covering pH from 4 to 8 established as described previously [32].

Vacuolar pH was measured with BCECF AM, a pH-sensitive fluorophore that accumulates in the yeast vacuole [32]. Strains were grown to mid log phase (OD ∼1) in SC medium. Cells were collected by centrifugation and incubated in SC containing 50 mM BCECF AM for 25 min. The cells were washed twice, suspended in SC to OD 2 and transferred to a 96-well plate. Fluorescence emission at 520 nm was measured in triplicate with excitation at 485 nm and 450 nm in a Fluostar Optima plate reader. Vacuolar pH was calculated based on the ratio of emission at 520 nm at dual excitations of 485 nm and 410 nm against a calibration curve covering pH from 4 to 8.5.

### Proton efflux assay

Proton efflux was assayed in wild-type and *erg24Δ* cells as described previously [55]. Briefly, cells were grown to ∼OD 1 in YPD. 1.5×10^8^ cells were pelleted by centrifugation and washed twice with distilled water. The washed cell pellet was resuspended in distilled water and placed on ice for 2–3 h. Just prior to use, the cells were centrifuged and resuspended in 3 ml of water. Once a stable pH base line was established, glucose was added to 2% to initiate proton efflux.

### Staining of *C. albicans* cells with quinacrine and FM4-64

Log phase cells of *C. albicans* (SC5314) were diluted to OD 0.025 and grown in YPD with or without fluconazole (1 µg/ml) for 5 hours. FM4-64 was added to 5 µM and the cultures were grown for another 30 min. Cells were washed twice in YPD, suspended in YPD with or without fluconazole, and shaken for 20 min. Quinacrine was then added to 100 µg/ml and the cultures were shaken for another 5 min. The cells were collected and washed twice in YPD and examined by fluorescence microscopy immediately.

### Ethics statement

All animal experimentation was conducted following the United States Public Health Service guidelines for housing and care of laboratory animals and performed in accordance with Institutional regulations after pertinent review and approval by the Institutional Animal Care and Use Committee of the University of Medicine and Dentistry of New Jersey.

### Murine candidiasis model and antifungal assessment

Female BALB/c mice (age, 6 to 8 weeks; weight, 17 to 20 g) (Charles River Laboratories, Wilmington, MA) were used throughout the experiments. The mice were housed in micro-isolator cages with five animals per group and had access to food and water ad libitum. All animal experiments were conducted in the PHRI-UMDNJ Research Animal Facility. Disseminated infection with *C. albicans* (ATCC 36082) was induced by injection of 5.0×10^5^ blastoconidia (∼5 times the median lethal dose) in 0.1 ml of sterile saline via the lateral tail vein of female BALB/c mice. Therapy was initiated 3 hours after challenge. Mice were treated with vehicle, Fluconazole (LKT Laboratories Inc., St. Paul, MN) (0.1 to 60 mg/kg of body weight/dose), Amiodarone (Henry Schein Animal Health, Melville, NY) (5.0 or 25 mg/kg of body weight/dose) or Fluconazole (0.1 to 60 mg/kg of body weight/dose) + Amiodarone (5.0 or 25 mg/kg of body weight/dose) administered intraperitoneally once a day for a total of 4 days. On day 4 post-infection, kidneys from euthanized mice (5 per group, unless specified) were aseptically removed, weighed, and homogenized in sterile saline using an IKA Works ULTRA TURRAX Tube Disperser Workstation (IKA Works Inc., Wilmington, NC). Serial dilutions of homogenized kidneys were plated onto YPD agar plates containing chloramphenicol (70 µg/ml) and ampicillin (50µg/ml) to eliminate bacterial cross-contamination. Culture plates were incubated at 30°C for 48 h, after which the CFU were counted and the number of CFU per gram of tissue was calculated. The method was sensitive for detection of ≥10 CFU/g. The culture-negative plates were counted as having 0 CFU/g.

### Statistics

The association of *C. albicans* proliferation with treatment group was assessed using multiple linear regression of log-transformed CFU counts. Preliminary model fit analysis was conducted to confirm that a parsimonious model which included dose-response slopes for AMD and FLZ, and a term for synergistic effect of dual drug treatment fit the data as well as the saturated model, where each of the nine treatment and dosage combinations were independent predictors (likelihood ratio test p = 0.47). The regression coefficients for drug dose are interpreted as slopes of the dose response curves. For the synergistic term, the exponentiated coefficient is the ratio of geometric means of the CFU for any given dose of each drug when the other drug is present versus when the other drug is absent. The significance of the association of CFU with predictors was assessed using Wald t-statistics for the regression coefficients.

## Supporting Information

Figure S1 Sterol profiles of *upc2-1* cells treated with fluconazole and ergosterol. Total sterol from control WYP361 (*upc2-1*) cells (A), cells treated with fluconazole (100 µg/ml) for 6 hours (B), and those treated with fluconazole (100 µg/ml) and ergosterol (50 µM) together for 6 hours (C) was extracted and analyzed by gas chromatography. Cholesterol was added to the samples to normalize extraction efficiency. Ergosterol and lanosterol were identified with standards. A major sterol derivative, likely 14-methyl 3,6-diol, is marked with an asterisk. Analysis was done in triplicates for each treatment and a representative chromatogram is shown. (D) Quantification of ergosterol, lanosterol and the major sterol derivative. Averages of relative amounts and standard deviations were calculated from triplicate samples and plotted.
(0.86 MB TIF)
