# Supplementary figures and images for: Requirement for Ergosterol in V-ATPase Function Underlies Antifungal Activity of Azole Drugs
Source: PLoS Pathog. 2010 Jun 3;6(6):e1000939. doi: 10.1371/journal.ppat.1000939 (PMC2880581; doi:10.1371/journal.ppat.1000939)

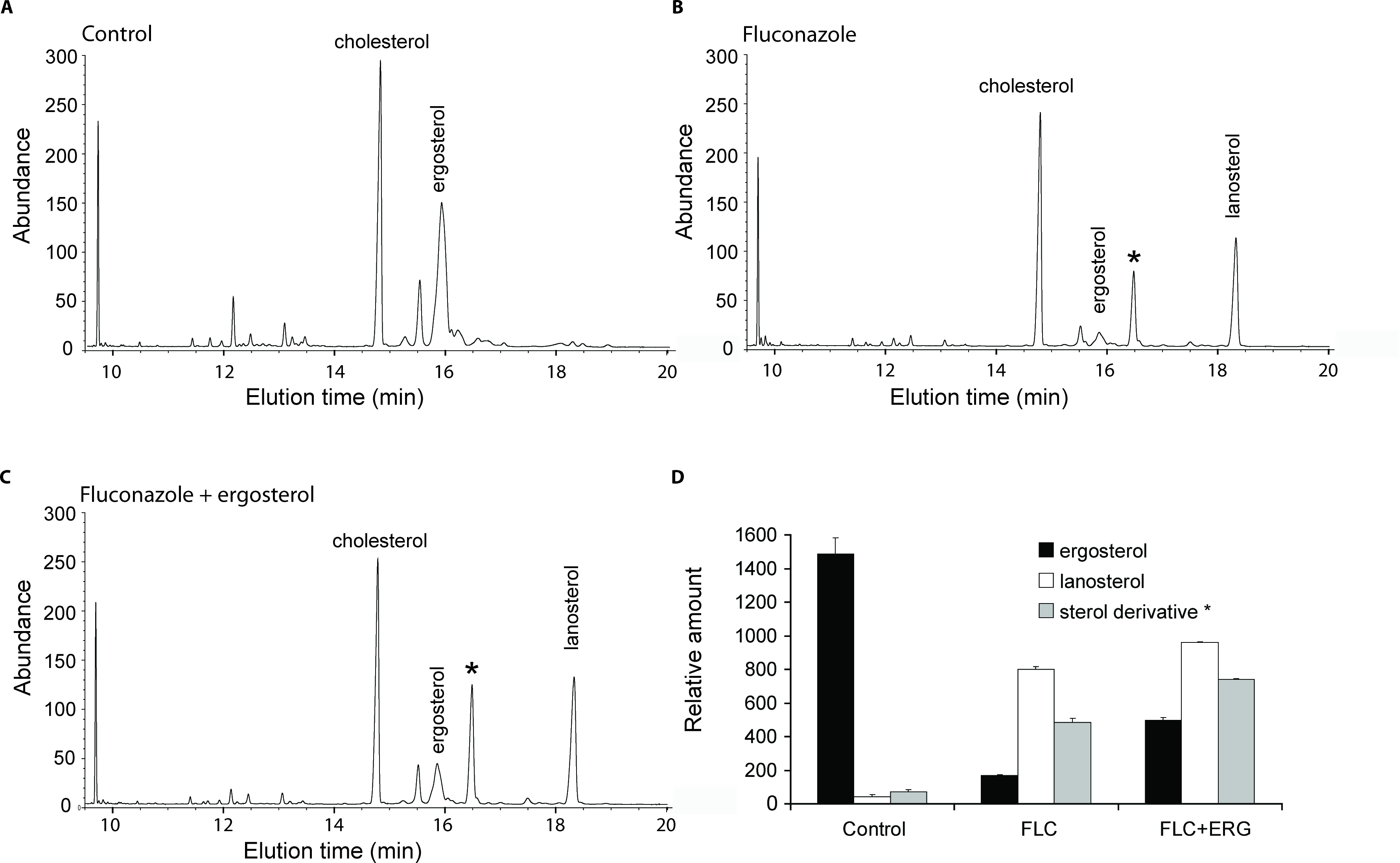

Supplement: Figure S1 — Sterol profiles of upc2-1 cells treated with fluconazole and ergosterol. Total sterol from control WYP361 (upc2-1) cells (A), cells treated with fluconazole (100 µg/ml) for 6 hours (B), and those treated with fluconazole (100 µg/ml) and ergosterol (50 µM) together for 6 hours (C) was extracted and analyzed by gas chromatography. Cholesterol was added to the samples to normalize extraction efficiency. Ergosterol and lanosterol were identified with standards. A major sterol derivative, likely 14-methyl 3,6-diol, is marked with an asterisk. Analysis was done in triplicates for each treatment and a representative chromatogram is shown. (D) Quantification of ergosterol, lanosterol and the major sterol derivative. Averages of relative amounts and standard deviations were calculated from triplicate samples and plotted. (0.86 MB TIF) [file ppat.1000939.s001.tif]
